# The expression pattern of pyruvate dehydrogenase kinases predicts prognosis and correlates with immune exhaustion in clear cell renal cell carcinoma

**DOI:** 10.1038/s41598-023-34087-x

**Published:** 2023-05-05

**Authors:** Caroline E. Nunes-Xavier, Maite Emaldi, Janire Mingo, Tove Øyjord, Gunhild M. Mælandsmo, Øystein Fodstad, Peio Errarte, Gorka Larrinaga, Roberto Llarena, José I. López, Rafael Pulido

**Affiliations:** 1 Biomarkers in Cancer Unit, Biocruces Bizkaia Health Research Institute, Barakaldo, Spain; 2 Department of Tumor Biology, Institute for Cancer Research, Oslo University Hospital Radiumhospitalet, Oslo, Norway; 3 University of Tromsø - The Arctic University of Norway, Tromsø, Norway; 4 Department of Nursing, Faculty of Medicine and Nursing, University of the Basque Country UPV/EHU, Leioa, Spain; 5 Department of Physiology, Faculty of Medicine and Nursing, University of the Basque Country UPV/EHU, Leioa, Spain; 6 Department of Urology, Cruces University Hospital, Barakaldo, Spain; 7 Ikerbasque, Basque Foundation for Science, Bilbao, Spain

**Keywords:** Cancer metabolism, Renal cancer

## Abstract

Renal cancer cells constitute a paradigm of tumor cells with a glycolytic reprogramming which drives metabolic alterations favouring cell survival and transformation. We studied the expression and activity of pyruvate dehydrogenase kinases (PDK1-4), key enzymes of the energy metabolism, in renal cancer cells. We analysed the expression, subcellular distribution and clinicopathological correlations of PDK1-4 by immunohistochemistry of tumor tissue microarray samples from a cohort of 96 clear cell renal cell carcinoma (ccRCC) patients. Gene expression analysis was performed on whole tumor tissue sections of a subset of ccRCC samples. PDK2 and PDK3 protein expression in tumor cells correlated with lower patient overall survival, whereas PDK1 protein expression correlated with higher patient survival. Gene expression analysis revealed molecular association of PDK2 and PDK3 expression with PI3K signalling pathway, as well as with T cell infiltration and exhausted CD8 T cells. Inhibition of PDK by dichloroacetate in human renal cancer cell lines resulted in lower cell viability, which was accompanied by an increase in pAKT. Together, our findings suggest a differential role for PDK enzymes in ccRCC progression, and highlight PDK as actionable metabolic proteins in relation with PI3K signalling and exhausted CD8 T cells in ccRCC.

## Introduction

The tight connection between cell growth-related signalling pathways and metabolic homeostasis pathways has been disclosed in the last years as one of the hallmarks of cancer, making metabolic enzymes potential targets for cancer therapeutics^1–3^. A landmark example is the case of metabolic kinases, whose catalytic activities have been found to go beyond metabolite phosphorylation and are also physiologically used to phosphorylate protein substrates, regulating essential cell growth functions^4^.

Renal cancers display a high extent of metabolic alterations, mainly related with cell metabolic reprogramming towards glycolytic degradation of glucose in the presence of oxygen (Warburg effect) and de novo synthesis of fatty acids, which facilitate the synthesis of biomolecules required by the tumor cells to keep their rapid growth and division^5,6^. This reprogramming is shared by other types of cancer and it is commonly associated with the activation of the PI3K/AKT/PTEN/mTOR survival pathway^7^. Several hereditary syndromes linked to renal cancer are characterized by mutations in genes coding for key metabolic enzymes, such as fumarate hydratase (FH) and succinate dehydrogenase B (SDHB), and defects in these enzymes associate with malignancy and poor prognosis in renal cell carcinoma (RCC)^8,9^. This makes renal cancers a paradigm in the interface between metabolic- and oncogenic-diseases^10,11^. RCC is the most frequent renal neoplasm (90–95% of renal cancer cases), with the clear cell renal carcinomas (ccRCC) being the most abundant and of worse prognosis subtype. About 5% of ccRCC is linked to hereditary cancer caused by mutations in the tumor suppressor Von Hippel-Lindau (VHL), a promoter of the degradation of the transcription factor HIF1^12,13^. In sporadic ccRCC, genetic inactivation of VHL is the most common alteration, although VHL mutations are infrequent in other cancers^14,15^.

Intratumor heterogeneity at histopathological, immunohistochemical, and genetic levels is an intrinsic property of renal cancers, and it is a consequence of the temporal clonal and sub-clonal evolution of malignant cells, which impacts negatively in the efficacy of the therapeutic treatments^16–18^. The global genomic analysis of ccRCC performed by the TRACERx Renal Consortium has revealed the existence of 7 subtypes of tumor evolution and 2 subtypes of metastatic dissemination^19–21^. Recently, it has been described in RCC the existence of metabolic intratumor heterogeneity related with pyruvate metabolism, and pharmacologic interventions in this pathway have been proposed as novel therapeutic approaches^22^. The enzyme pyruvate dehydrogenase (PDH) is an essential component of the oxidative versus non-oxidative energy cellular metabolism, playing important roles in carcinogenesis^23–25^. PDH exists as an enzymatic multimolecular complex formed by the assembly of three catalytic (E1, E2, and E3) and three regulatory subunits. The pyruvate dehydrogenase kinases (PDK; four genes: *PDK1-4*) are major regulatory subunits of the PDH complex, since phosphorylation of E1 catalytic subunit by PDK inhibits PDH activity^26^. Association of expression of distinct PDK with poor prognosis and resistance to anti-cancer therapies is documented, and PDK pharmacological inhibition (which results in PDH activation) constitutes a potentially operative therapy in several cancer types, including renal cancer^23,24,27^. The selective involvement of distinct PDK in cancer seems to be tissue-specific^28,29^, making relevant to investigate the individual expression and function of PDK1-4 in relation with the growth and survival properties of renal cancer cells.

The PI3K/PTEN/AKT/mTOR pathway is frequently altered, both mechanistically and mutationally, in renal cancer^7,30^. *PIK3CA* and *MTOR* genes show gain-of-function mutations in about 2.5 and 6%, respectively, of renal tumors (COSMIC database), and the inhibition of the major effectors of the pathway (PI3K catalytic subunits, AKT, mTOR) is under intense exploration in clinical trials. In fact, mTOR inhibitory drugs are already approved for the treatment of advanced RCC^31–33^. The PTEN tumor suppressor is the physiologic inactivator of the PI3K pathway^34^. Accordingly, *PTEN* gene loss-of-function mutations are found in about 4% of renal tumors (COSMIC database). The inhibition of PI3K, AKT, or mTOR frequently courses with the reactivation of other oncogenic components of the pathway in cancer cells^35^, making important the identification and validation of alternative therapeutic targets.

In this study, we have explored the expression and functional relation with the PI3K pathway of the energy metabolism PDK1-4 enzymes in renal cancer cells. Our results suggest a differential involvement of the distinct PDK in renal tumorigenesis, and provide relevant information on novel onco-metabolic therapeutically actionable pathways in renal cancer.

## Results

**Figure 1:**
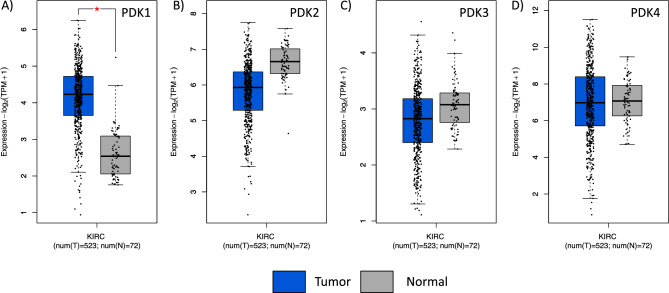
mRNA expression of PDK1-4 in normal kidney and in ccRCC. Box plots for PDK1 (**A**), PDK2 (**B**), PDK3 (**C**) and PDK4 (**D**) showing mRNA expression in ccRCC tumor tissue (shown in blue) in comparison to normal kidney tissue (in grey). Data is represented in a logarithmic scale (Log2) and obtained from 523 tumor samples and 72 normal tissue samples (KIRC, TCGA). Statistically significant results (p < 0.01) are marked with an asterisk.

**Figure 2:**
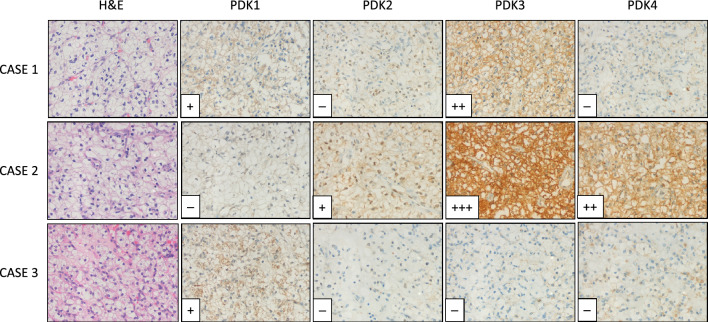
Protein expression of PDK1-4 in ccRCC tumors. Representative immunohistochemical staining of PDK1-4 protein expression in three representative ccRCC patient samples. H&E, Hematoxylin and Eosin staining. Case 1, positive staining of PDK1 and PDK3, and negative staining of PDK2 and PDK4. Case 2, moderate and high positive staining of PDK2, PDK3 and PDK4, and negative staining of PDK1. Case 3, positive staining of PDK1, and negative staining of PDK2, PDK3 and PDK4. Magnification: × 250.

**Figure 3:**
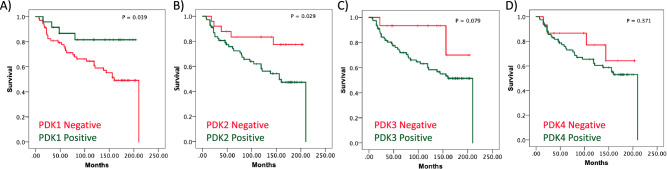
Kaplan–Meier survival curves of ccRCC patients according to PDK1-4 tumor expression. Kaplan–Meier curves of overall survival based on PDK1 (**A**), PDK2 (**B**), PDK3 (**C**), and PDK4 (**D**) protein expression in the ccRCC cohort.

In silico comparative analysis of the expression of *PDK1-4* genes in ccRCC shows distinct relative mRNA expression levels for these genes. A significant increase in PDK1 mRNA is observed when ccRCC is compared with normal tissue, whereas PDK2, PDK3 and PDK4 mRNA did not display significant changes (Fig. 1). To gain further insights into the contribution of the distinct PDK proteins in ccRCC, we evaluated by IHC the expression of PDK1-4 in tumor samples from a retrospective cohort of 96 ccRCC patients (Table 1). FFPE samples from two representative tumor areas of each case were included in TMA for analysis, and expression was scored as negative (when low or no staining) or positive (when moderate and high staining). Representative immunostaining images are shown in Fig. 2. We observed heterogeneous expression of the distinct PDK proteins. PDK1, PDK3, and PDK4 displayed a major cytoplasmic localization, whereas PDK2 showed a predominantly nuclear localization. Significant negative correlations were found of PDK1 expression with tumor diameter (p = 0.000) and higher overall survival (p = 0.010). Importantly, we found a significant positive correlation of PDK2 and PDK3 immunostaining with lower overall survival (p = 0.021 and p = 0.022), and a positive correlation of PDK3 expression with higher tumor grade (p = 0.038). We did not find correlations of PDK4 expression with any clinical parameter (Table 1). Kaplan–Meier curves for survival time and PDK1-4 expression showed differential significant correlations for PDK1 and PDK2 protein expression (log-rank test, p = 0.039 and p = 0.029 respectively) (Fig. 3). Patients with tumors positive for PDK1 expression had a higher survival probability than those with tumors negative for PDK1 expression, whereas the opposite correlation was found for PDK2 expression (Fig. 3).

**Table 1:** Correlation between clinical and pathological variables and PDK1-4 protein expression in clear cell renal cell carcinoma.

Characteristic ccRCC	PDK1			PDK2			PDK3			PDK4		
		PDK1 low	PDK1 high		PDK2 low	PDK2 high		PDK3 low	PDK3 high		PDK4 low	PDK4 high
Patients	N = 93	(N = 69)	(N = 24)	N = 94	(N = 25)	(N = 69)	N = 93	(N = 15)	(N = 78)	N = 93	(N = 15)	(N = 78)
Median follow-up time		R = 0.045/P = 0.645			R = − 0.182/P = 0.234			R = − 0.011/P = 0.874			R = − 0.045/P = 0.322	
Months	120.0	119.0	130.0	119.5	158.0	99.0	119.0	120.0	119.0	119.0	120.0	119.0
Median age at surgery		R = − 0.011/P = 0.129			R = − 0.001/P = 0.333			R = − 0.173/P = 0.656			R = − 0.005/P = 0.323	
Years	72	71	75.5	73	69	73	73	77	71.5	73	75	73
Sex		R = 0.123/P = 0.235			R = 0.128/P = 0.214			R = − 0.068/P = 0.512			R = 0.196/P = 0.059	
Female	24	20	4	25	9	16	25	3	22	25	7	18
Male	69	49	20	69	16	53	68	12	56	68	8	60
Age at surgery		R = 0.029/P = 0.782			R = 0.080/P = 0.441			R = − 0.195/P = 0.060			R = 0.042/P = 0.685	
≤ 70 years	41	31	10	39	12	27	39	3	36	39	7	32
> 70 years	52	38	14	55	13	42	54	12	42	54	8	46
Grade*		R = − 0.186/P = 0.074			R = 0.007/P = 0.946			**R = 0.216/P = 0.038**			R = 0.033/P = 0.751	
Low	59	40	19	59	16	43	58	**13**	**45**	58	10	48
High	33	28	5	34	9	25	34	**2**	**32**	34	5	29
Stage**		R = − 0.197/P = 0.058			R = 0.064/P = 0.537			R = 0.124/P = 0.232			R = 0.000/P = 1.000	
Low	63	43	20	63	18	45	62	12	50	62	10	52
High	30	26	4	31	7	24	31	3	28	31	5	26
Diameter***		**R = − 0.400/P = 0.000**			R = 0.126/P = 0.220			R = 0.186/P = 0.073			R = 0.000/P = 1.000	
≤ 4 cm	32	**16**	**16**	32	11	21	31	8	23	31	5	26
> 4 cm	61	**53**	**8**	62	14	48	62	7	55	62	10	52
Survival		**R = − 0.267/P = 0.010**			**R = 0.239/P = 0.021**			**R = 0.237/P = 0.022**			R = 0.118/P = 0.257	
Alive	57	**37**	**20**	57	**20**	**37**	56	**13**	**43**	56	11	45
Dead	36	**32**	**4**	37	**5**	**32**	37	**2**	**35**	37	4	33

Significant values are in bold.

*Fuhrman’s grade, low (G1/2) vs. high (G3/4); **AJCC 2010 staging low (pT1/2) vs. high (≥ pT3); ***Tumor diameter, small (≤ 4 cm) vs. large (> 4 cm).

Pearson’s correlation, R; p value, P.

**Figure 4:**
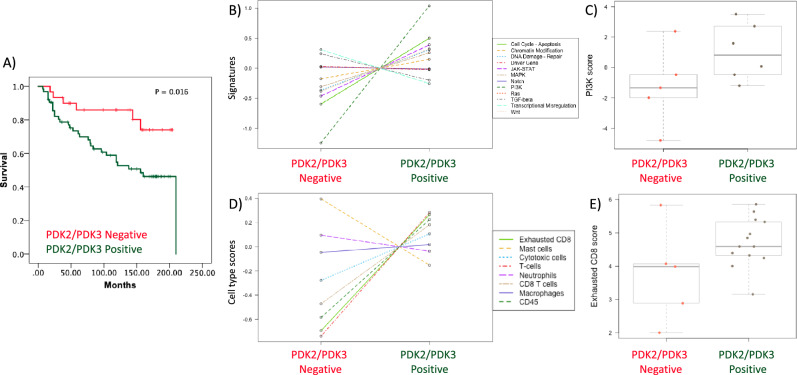
Molecular analysis of ccRCC patients according to PDK2 and PDK3 tumor expression. (**A**) Kaplan–Meier curves of overall survival based on PDK2/PDK3 expression in the ccRCC cohort. Patients with high PDK2/PDK3 protein expression and patients with low PDK2/PDK3 protein expression had significantly different time to recurrence (p = 0.016). (**B**) PanCancer Pathway signature in ccRCC tumor samples. Note PI3K pathway as most differentially expressed signalling pathway in high PDK2/PDK3 protein expression vs low PDK2 and PDK3 protein expression tumors. (**C**) Box plot of PI3K pathway score in high PDK2 and PDK3 protein expression vs low PDK2 and PDK3 protein expression tumors. (**B**, **C**) Data is represented in a logarithmic scale (Log2) and obtained from 11 ccRCC tumor samples. (**D**) Cell type score in ccRCC tumor samples using Immune Exhaustion panel. Note CD8 T cell exhaustion cell type as one of the higher enrichments in high PDK2/PDK3 protein expression vs low PDK2/PDK3 protein expression tumors. (**E**) Box plot of exhausted CD8 score in high PDK2/PDK3 protein expression vs low PDK2/PDK3 protein expression tumors. (**D**, **E**) Data is represented in a logarithmic scale (Log2) and obtained from 18 ccRCC tumor samples. Statistically significant results (p < 0.05) are marked with an asterisk.

Of interest, we found an increased correlation with overall survival when we compared double positive PDK2/PDK3 expression (lower survival) with double negative PDK2/PDK3 expression (higher survival) (p = 0.007, R = 0.279). Furthermore, Kaplan–Meier survival time analysis of double positive PDK2/PDK3 expression *vs* double negative expression showed a significant increase in the differences in survival time (log-rank test, p = 0.016) (Fig. 4A).

Next, we performed experiments of molecular gene profiling analysis using PanCancer Pathway and Immune Exhaustion panels (nCounter, NanoString) on RNA isolated from tumor areas on whole tissue sections. Differentially expressed genes in PDK2/PDK3 positive tumors, as compared to the baseline PDK2/PDK3 negative tumors, are shown in volcano plots (Supplementary Fig. 1A,B). Of the more differentially expressed genes from the PanCancer Pathway panel, we observed significant higher expression of FN1 (p = 0.007), MMP7 (p = 0.0272), and COL1A1 (p = 0.0252) genes; and lower expression of EPOR (p = 0.007) (Supplementary Table 1) in PDK2/PDK3 positive tumors compared to PDK2/PDK3 negative tumors. In the Immune Exhaustion panel, we observed significant higher expression of UHRF1 (p = 0.002) and VAV3 (p = 0.021), and lower expression of RGS16 (p = 0.023) and PLCG2 (p = 2.01e-05) genes (Supplementary Table 2). In silico analysis from ccRCC TCGA dataset confirmed significant positive correlations between FN1, MMP7, COL1A1 and UHRF1 with PDK3 expression, and between VAV3 with PDK2 and PDK3 expression, and a negative correlation between EPOR and PDK3 expression, and RGS16 with PDK2 (Supplementary Figs. 2 and 3). Pathway analysis showed the higher pathway scores with the PI3K signature signalling pathway in PDK2/PDK3 positive tumors (p < 0.05) (Fig. 4B), indicating a positive correlation of PDK2/PDK3 expression with PI3K signalling pathway (Fig. 4C). Furthermore, cell type deconvolution analysis revealed increased T cells, CD8 cells and increased exhausted CD8 T cells in the double positive PDK2/PDK3 tumors (Fig. 4D,E).

**Figure 5:**
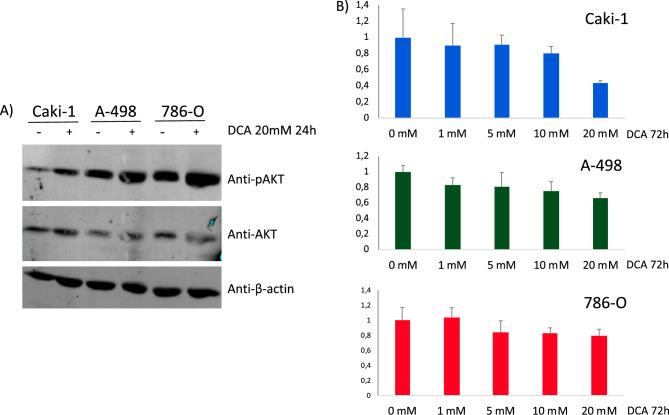
(**A**) Immunoblot of pAKT, AKT and β-actin in ccRCC cells treated with PDK inhibitor DCA (20 mM for 24 h). Cropped blots are displayed. (**B**) Proliferation of Caki-1, A-498, and 786-O ccRCC cells, treated with dichloroacetate (DCA). Cell viability is shown, as determined by MTS analysis, after 72 h in the presence of DCA (1–20 mM for 72 h).

To analyse the functional role of PDK in renal cancer cells, we monitored phospho-AKT (pAKT) status and cell proliferation/viability on Caki-1, A-498, and 786-O ccRCC cell lines, using the pan-PDK inhibitor dichloroacetate (DCA)^36^. An increase in pAKT content was observed in ccRCC cells upon PDK inhibition by DCA (Fig. 5A). In addition, DCA treatment caused a dose response growth inhibitory effect on the renal cancer cell lines, as shown by MTS assay (Fig. 5B). These results suggest a role for PDK in the regulation of cell growth and viability of ccRCC cells, likely in coordination with PI3K signalling pathway alterations.

## Discussion

Different types of renal cancer share in common an association with reprogramming of glucose and fatty acid metabolism and the tricarboxylic acid (TCA) cycle^6,37^. Metabolic tumor susceptibilities present in the particular environment of renal cancer cells grant opportunities for novel treatment paradigms which are being tested alone or in combinations to targeted or immune-based strategies^38^. Our results suggest that inhibition of PDK could have potential therapeutic benefits in ccRCC.

We found that higher PDK1 protein expression associated with increased 10-year overall survival. In contrast, Baumunk et al., found that lower PDK1 mRNA expression associated with better outcome^39^. PDK1 increased expression in ccRCC tumor tissues has been documented previously both at mRNA and protein level^27,39,40^. Our in silico analysis of ccRCC cells and normal kidney tissue showed higher expression of PDK1 mRNA in ccRCC tumors, whereas no significant differences were found for PDK2, PDK3 and PDK4 mRNA expression in normal tissue vs ccRCC. Together, this suggests a potential selective involvement of specific PDK in renal cancer. PDK1 has been previously described as a target of HIF1α (*HIF1A* gene) transcriptional metabolic reprogramming^41^. In silico correlation analysis between PDK1–4 and HIF1α expression in ccRCC showed significant correlations with PDK1, PDK3 and PDK4 expression (p < 0.005), but not with PDK2 (Supplementary Fig. 4A). On the other hand, HIF2α (*EPAS1* gene) correlation analysis showed statistical correlation with all PDK (Supplementary Fig. 4B). In addition, PDK3 protein overexpression in ccRCC samples has been reported to correlate with VHL non-mutated status^42^. The connection of HIF-induced metabolic reprogramming of renal cancer cells with selective expression of PDK1-4 deserves further analysis.

We have also found that positive PDK2 and PDK3 expression at the time of surgery could predict poorer survival of ccRCC patients. Whether high expression of PDK2 and PDK3 in ccRCC may have oncogenic consequences or may be an effect of early oncogenic metabolic reprogramming is currently unknown. We observed a difference in the subcellular localization of PDK2 and PDK3 proteins in ccRCC specimens, with PDK2 displaying enriched nuclear localization, similar to previously observed patterns in prostate cancer^43^. This could indicate a differential spatial role of the TCA cycle in cancer^44,45^. The understanding of the nuclear TCA cycle is still largely unclear, but seems to be important in development^46^. The specific role of PDK nuclear localization in tumor progression is still unknown, and requires dedicated studies.

DCA-mediated inhibition of PDK manifests anti-oncogenic properties in cancer cells, including ccRCC cells, in association with inhibition of HIF transcriptional activity^27,47^. Our results show growth inhibition by DCA on three ccRCC cell lines, suggesting that poor prognosis-PDK2/PDK3 high expression ccRCC cases could benefit upon treatment with DCA analogues. DCA also increased pAKT content in ccRCC cells, reinforcing the correlation observed between PDK2/PDK3 and PI3K signalling pathway. Nevertheless, since DCA also inhibits PDK1^48^, whose expression was associated with better prognosis in our cohort, a differential contribution of PDK1-4 inhibition in ccRCC growth inhibition is expected, as it has also been proposed for other cancers^43,49^.

Our molecular gene profiling analysis suggests differences in the tumor microenvironment in groups of ccRCC tumors depending on PDK2/PDK3 expression. We found an overall increase in T cell infiltration and CD8 exhausted T cells in PDK2/PDK3 positive ccRCC. This is in consistence with previous T cell landscape studies of ccRCC, which revealed higher proportion of exhausted CD8 T cell in advanced ccRCC^50^, and reinforces the importance of the crosstalk between metabolism and T cell exhaustion in ccRCC^51,52^. Relevant to this, lactate administration in mice was recently associated with CD8 T cell stemness and increased anti-tumor immunity^53^. This may be relevant in novel strategies to improve metabolic function of ccRCC CD8 T cells, which may promote the anti-tumor immune response. Since our study has the limitation of a small number of samples, further studies in larger cohorts should be made to confirm these findings.

In conclusion, our results show differential expression patterns of pyruvate dehydrogenase kinases in ccRCC. Expression of PDK2 and PDK3 predicts poor prognosis and correlates with immune exhaustion. We speculate that interference with specific PDK activities could have therapeutic benefits in groups of ccRCC patients.

## Materials and methods

### Cell culture and cell proliferation

Human clear cell renal carcinoma Caki-1 (Caki-1ATCC® HTB46™), 786-O (ATCC® CRL1932™), and A-498 (ATCC® HTB 44™) cells (purchased from American Type Culture Collection (ATCC) were cultured in McCoy5A, RPMI-1640 and EMEM medium (Lonza), respectively, supplemented with 10% FBS. All experiments were performed with mycoplasma-free cells. All media were supplemented with 1% l-Glutamine and 1% penicillin/streptomycin (Lonza). Cells were incubated at 37 °C and 5% CO_2_.

To assess cell proliferation/viability of Caki-1, A-498 and 786-O cells, 3500, 1500, and 1500 cells, respectively, were plated per well in 96-well culture plates. A day after plating the cells, different concentrations of dichloroacetate (DCA; Sigma Aldrich) or vehicle were added. Cell proliferation was measured with the CellTiter 96® AQueous One Solution Cell Proliferation Assay Kit (MTS Assay, Promega) in 96-well plates, and absorbance was measured at 490 nm using microplate reader 72 h post treatment (BioRad).

### Clinical material and tumor samples

The renal cancer cohort consisted of formalin-fixed paraffin-embedded (FFPE) tumor samples from 96 clear cell renal cell carcinomas (ccRCC) patients that underwent nephrectomy at University Hospital Cruces between 1997 and 2001. The median age was 73 years, median follow-up of the patients was 119.5 months, and the median diameter of the tumors was 5.25 cm. An experienced pathologist (JIL) selected tumor areas with well-preserved tissue representative of the whole tumor from FFPE tissue blocks from these patients, and tissue microarray (TMA) blocks were made from these areas. 4 µm sections were made from the TMA blocks, one of which was stained with hematoxylin and eosin to verify the presence of tumor content. Fuhrman’s grade^54^ and 2010 AJCC Staging system^55^ assigning was performed on hematoxylin & eosin sections from tumor samples obtained following standard protocols. Follow-up was obtained from the clinical histories and was closed 31st December 2014, with more than 10-year overall survival. Renal cancer cohort has been previously described in reference^56^.

### Immunohistochemistry and scoring

Immunohistochemistry (IHC) was carried out using the following primary antibodies: PDK1 (Sigma Aldrich, HPA027376, dilution: 1:20), PDK2 (Sigma Aldrich, HPA008287, dilution 1:25), PDK3 (Sigma Aldrich, HPA046583, dilution 1:50), and PDK4 (Sigma Aldrich, HPA056731, dilution 1:100) antibodies. Antigen retrieval was performed at pH 6 or pH 9 using PT link system (Agilent Technologies). IHC immunostainings were performed in automated immunostainers (EnVision FLEX, Dako Autostainer Plus; Dako, and BenchMark Ultra, Ventana Medical Systems). Antibodies were incubated for 30 min, followed by secondary antibody incubation for 15 min using goat anti-mouse or anti-rabbit Ig/HRP secondary antibodies (Dako), FLEX/HPR for 20 min, FLEX DAB/Sub Chromo for 10 min, and finally counterstaining with hematoxylin. Immunostainings were evaluated by an experienced uropathologist (JIL) in tumor cells as negative (low/no staining) or positive (moderate/high staining). The analysis was performed using a Nikon Eclipse 80i microscope (Nikon).

### Gene expression analysis

The mRNA expression levels of PDK1, PDK2, PDK3 and PDK4 in ccRCC tumor tissue was analysed in comparison to normal tissues, or in correlation analysis, using KIRC normal and tumor data from TCGA database via online web server GEPIA (http://gepia.cancer-pku.cn)^57^.

A subset of 11 and 18 ccRCC patient samples (whole tissue sections) were selected for nCounter PanCancer Pathway and Immune Exhaustion panels, respectively (NanoString). Total RNA was extracted using Qiagen Allprep FFPE DNA/RNA extraction kit, and RNA quality was assessed with Bioanalyzer. NanoString nCounter analysis was performed at the Oslo University Hospital Genomics core facility. From the 11 ccRCC patient samples analysed by the PanCancer Pathway panel, 6 tumors from matching TMA displaying positive PDK2 and PDK3 immunostaining, and 5 tumors displaying negative PDK2 and PDK3 immunostaining. From the 18 ccRCC patient samples analysed by the Immune Exhaustion panel, 14 tumors displayed positive PDK2 and PDK3 immunostaining, and 5 tumors displayed negative PDK2 and PDK3 immunostaining, as monitored in the TMA. Raw RNA expression data were normalized and quality controlled, and analysis was performed using the nSolver version 4.0. Identified pathway and cell type scores were generated using nCounter data resource.

### Statistical analysis

Error bars in results represent ± standard deviation (S.D.). Cell data was analyzed by GraphPad Prism t Test Calculator, where significance was calculated using two-tailed student t-test. All experiments were performed at least twice, and results shown are from one representative experiment. The SPSS version 23 software (SPSS Inc., IBM) was used for statistical calculations of the clinical material. Pearson's chi-square test was used to correlate PDK1-4 expression to clinicopathologic parameters. The estimated survival curves were compared using the log‐rank test. Detection of significant statistical differentially expressed genes, pathway and cell type analysis was assessed using nSolver advanced analysis software 4.0, R version 3.3.2. (The R foundation of statistical analyses) and XQuartz software. For all the experiments, p value below 0.05 was considered statistically significant.

### Ethical statement

The study was performed in accordance with the Declaration of Helsinki. Ethical approval was obtained from Comité de Ética de la Investigación con medicamentos de Euskadi (CEIm-E) (number PI2016096 and PI2022085).

## Supplementary Information

Supplementary Information.

## Data Availability

All data generated in this study is included in the manuscript or in supplementary data. The full nCounter analysis generated datasets are available from the corresponding authors on reasonable request.
