# Author Correction: The expression pattern of pyruvate dehydrogenase kinases predicts prognosis and correlates with immune exhaustion in clear cell renal cell carcinoma

**DOI:** 10.1038/s41598-023-36328-5

**Published:** 2023-06-06

**Authors:** Caroline E. Nunes‑Xavier, Maite Emaldi, Janire Mingo, Tove Øyjord, Gunhild M. Mælandsmo, Øystein Fodstad, Peio Errarte, Gorka Larrinaga, Roberto Llarena, José I. López, Rafael Pulido

**Affiliations:** 1 Biomarkers in Cancer Unit, Biocruces Bizkaia Health Research Institute, Barakaldo, Spain; 2 Department of Tumor Biology, Institute for Cancer Research, Oslo University Hospital Radiumhospitalet, Oslo, Norway; 3 University of Tromsø - The Arctic University of Norway, Tromsø, Norway; 4 Department of Nursing, Faculty of Medicine and Nursing, University of the Basque Country UPV/EHU, Leioa, Spain; 5 Department of Physiology, Faculty of Medicine and Nursing, University of the Basque Country UPV/EHU, Leioa, Spain; 6 Department of Urology, Cruces University Hospital, Barakaldo, Spain; 7 Ikerbasque, Basque Foundation for Science, Bilbao, Spain

Correction to: *Scientific Reports* 10.1038/s41598-023-34087-x, published online 05 May 2023

The original version of this Article contained an error.

In the Materials and methods section, under the subheading ‘Immunohistochemistry and scoring’,

“Immunohistochemistry (IHC) was carried out using the following primary antibodies: PDK1 (Cell Signaling #3062, dilution: 1:120), PDK2 (Sigma Aldrich, HPA008287, dilution 1:25), PDK3 (Sigma Aldrich, HPA046583, dilution 1:50), and PDK4 (Sigma Aldrich, HPA056731, dilution 1:100) antibodies.”

now reads:

“Immunohistochemistry (IHC) was carried out using the following primary antibodies: PDK1 (Sigma Aldrich, HPA027376, dilution: 1:20), PDK2 (Sigma Aldrich, HPA008287, dilution 1:25), PDK3 (Sigma Aldrich, HPA046583, dilution 1:50), and PDK4 (Sigma Aldrich, HPA056731, dilution 1:100) antibodies.”

The original Article has been corrected.

