# Supplementary material for: The expression pattern of pyruvate dehydrogenase kinases predicts prognosis and correlates with immune exhaustion in clear cell renal cell carcinoma
Source: Sci Rep. 2023 May 5;13:7339. doi: 10.1038/s41598-023-34087-x (PMC10162970; doi:10.1038/s41598-023-34087-x)

SUPPLEMENTARY FIGURE LEGENDS

Supplementary Figure 1.

A) Volcano plot of differentially expressed genes from Pan Cancer Pathway panel of 11 ccRCC tumor samples when comparing PDK2/PDK3 positive versus PDK2/PDK3 negative tumors. B) Volcano plot of differentially expressed genes from Immune Exhaustion panel of 18 ccRCC tumor samples when comparing PDK2/PDK3 positive versus PDK2/PDK3 negative tumor.

Supplementary Figure 2.

In silico validation of correlation analysis of differentially expressed genes in PDK2 and PDK3 high tumors from nCounter Pan Cancer Panel. Correlation analysis of PDK2 and PDK3 mRNA expression with FN1 (A), MMP7 (B), COL1A1 (C) and EPOR (D) mRNA expression is shown. Data are from KIRC, TCGA.

Supplementary Figure 3.

In silico validation of correlation analysis of differentially expressed genes in PDK2 and PDK3 high tumors from nCounter Immune Exhaustion Panel. Correlation analysis of PDK2 and PDK3 mRNA expression with UHRF1 (A), VAV3 (B), RGS16 (C) and PLCG2 (D) mRNA expression is shown. Data are from KIRC, TCGA.

Supplementary Figure 4.

Correlation analysis of PDK1-4 mRN expression with HIF1α (*HIF1A*) (A) and HIF2α (*EPAS1*) (B) mRNA expression. Data are from KIRC, TCGA.

Supplementary Figure 1.

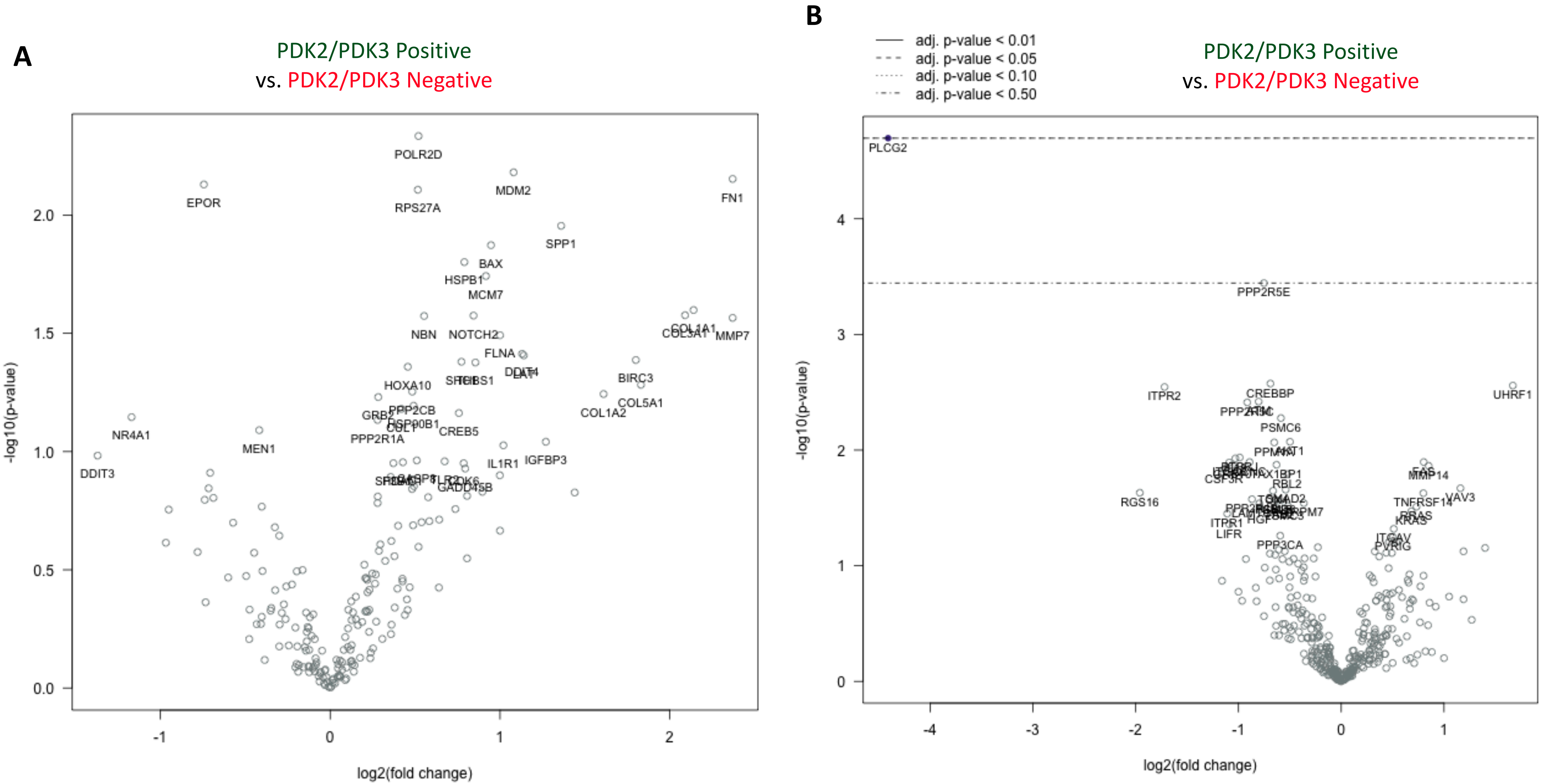

Supplementary Figure 2.

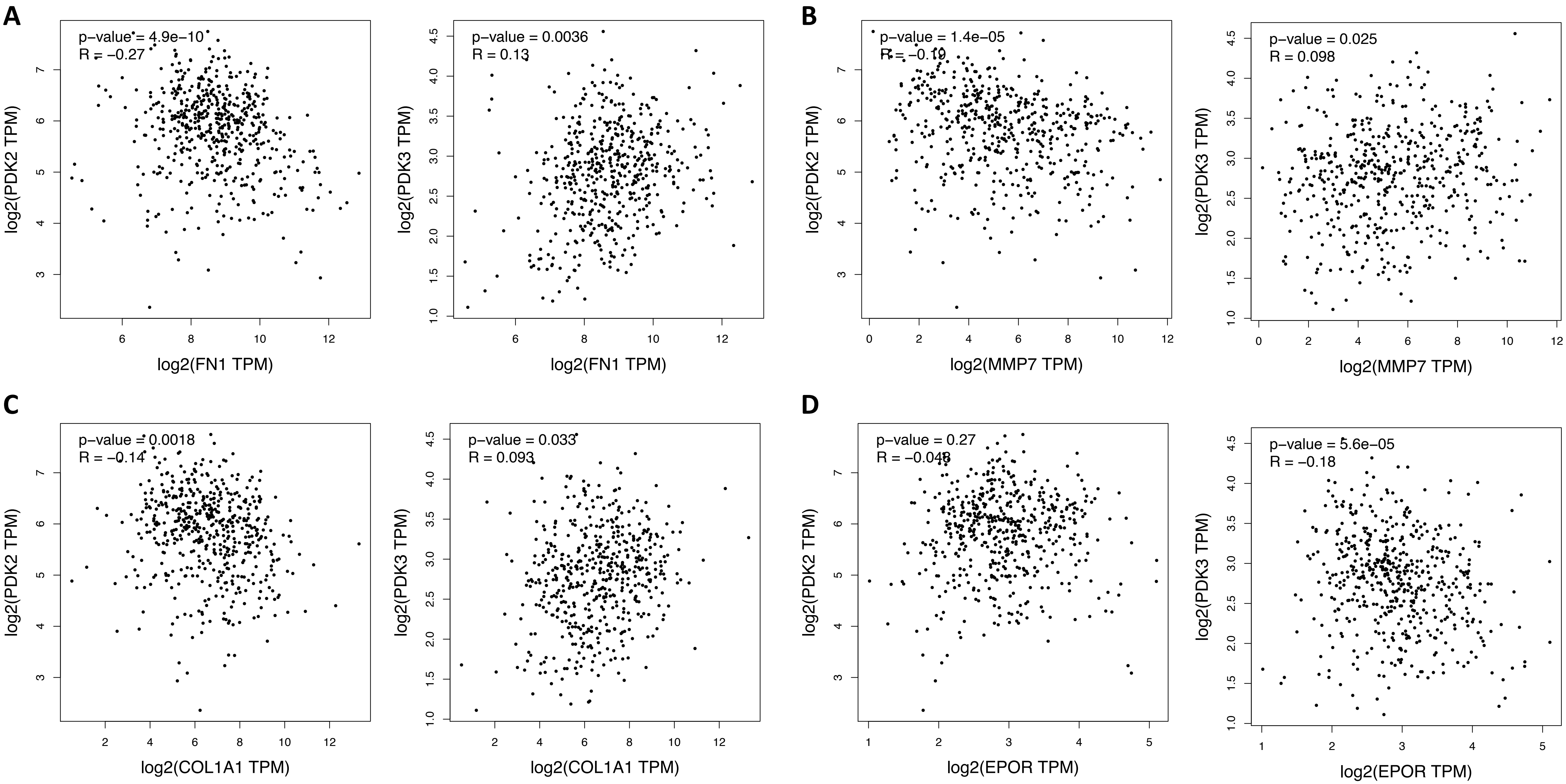

Supplementary Figure 3.

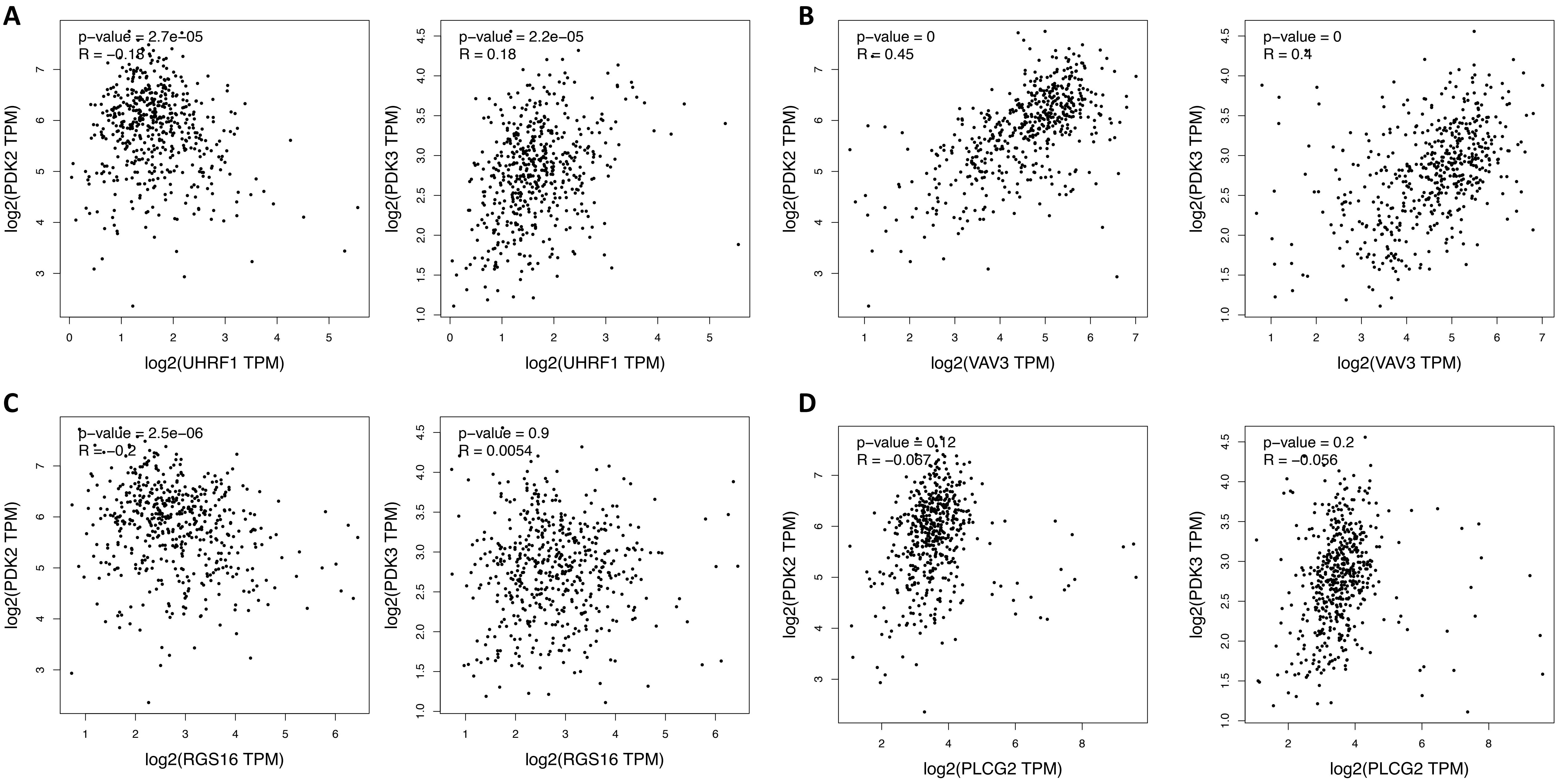

SUPPLEMENTARY TABLE 1. Differentilly Expressed Genes in PDK2/PDK3 positive versus PDK2/PDK3 negative tumors in nCounter PanCancer Pathway Panel

	Log2 fold change	std error (log2)	Lower confidence	Upper confidence	Linear fold change	Lower confidence	Upper confidence	P-value
**FN1-mRNA**	2.37	0.683	1.03	3.71	5.17	2.05	13.1	0.00703
**MMP7-mRNA**	2.37	0.9	0.607	4.13	5.17	1.52	17.6	0.0272
**COL1A1-mRNA**	2.14	0.799	0.575	3.71	4.41	1.49	13.1	0.0252
**COL3A1-mRNA**	2.09	0.787	0.543	3.63	4.24	1.46	12.4	0.0265
**BIRC3-mRNA**	1.8	0.757	0.32	3.29	3.49	1.25	9.77	0.041
**SPP1-mRNA**	1.36	0.427	0.524	2.2	2.57	1.44	4.59	0.0111
**LAT-mRNA**	1.14	0.475	0.214	2.07	2.21	1.16	4.21	0.0392
**DDIT4-mRNA**	1.13	0.467	0.215	2.05	2.19	1.16	4.13	0.0386
**MDM2-mRNA**	1.08	0.306	0.476	1.68	2.11	1.39	3.2	0.00659
**BAX-mRNA**	0.947	0.308	0.342	1.55	1.93	1.27	2.93	0.0134
**MCM7-mRNA**	0.917	0.318	0.294	1.54	1.89	1.23	2.91	0.0181
**THBS1-mRNA**	0.855	0.361	0.148	1.56	1.81	1.11	2.95	0.042
**NOTCH2-mRNA**	0.844	0.319	0.219	1.47	1.8	1.16	2.77	0.0266
**HSPB1-mRNA**	0.79	0.266	0.268	1.31	1.73	1.2	2.48	0.0158
**SHC1-mRNA**	0.773	0.326	0.135	1.41	1.71	1.1	2.66	0.0417
**NBN-mRNA**	0.553	0.209	0.143	0.963	1.47	1.1	1.95	0.0267
**POLR2D-mRNA**	0.52	0.139	0.247	0.793	1.43	1.19	1.73	0.00463
**RPS27A-mRNA**	0.516	0.152	0.219	0.814	1.43	1.16	1.76	0.00781
**HOXA10-mRNA**	0.457	0.195	0.0748	0.84	1.37	1.05	1.79	0.0438
**FLNA-mRNA**	1	0.397	0.227	1.78	2.01	1.17	3.44	0.0322
**EPOR-mRNA**	-0.744	0.216	-1.17	-0.32	0.597	0.445	0.801	0.00742

SUPPLEMENTARY TABLE 2. Differentilly Expressed Genes in PDK2/PDK3 positive versus PDK2/PDK3 negative tumors in nCounter Immune Exhaustion Panel

	Log2 fold	std error (log2)	Lower confidence	Upper confidence	Linear fold	Lower confidence	Upper confidence	P-value
**UHRF1-mRNA**	1.67	0.474	0.744	2.6	3.19	1.68	6.07	0.00277
**VAV3-mRNA**	1.16	0.455	0.269	2.05	2.24	1.2	4.15	0.0214
**MMP14-mRNA**	0.851	0.307	0.249	1.45	1.8	1.19	2.74	0.0137
**FAS-mRNA**	0.803	0.286	0.242	1.36	1.74	1.18	2.57	0.0127
**TNFRSF14-mRN/**	0.799	0.32	0.173	1.43	1.74	1.13	2.69	0.0236
**RRAS-mRNA**	0.732	0.309	0.126	1.34	1.66	1.09	2.53	0.0309
**KRAS-mRNA**	0.683	0.294	0.106	1.26	1.61	1.08	2.4	0.0338
**ITGAV-mRNA**	0.511	0.239	0.0431	0.978	1.42	1.03	1.97	0.048
**TRPM7-mRNA**	-0.364	0.152	-0.661	-0.0669	0.777	0.632	0.955	0.0288
**AKT1-mRNA**	-0.499	0.166	-0.825	-0.173	0.708	0.564	0.887	0.00848
**SMC3-mRNA**	-0.518	0.218	-0.946	-0.0901	0.698	0.519	0.939	0.0305
**RBL2-mRNA**	-0.526	0.196	-0.91	-0.143	0.694	0.532	0.906	0.0162
**SMAD2-mRNA**	-0.541	0.213	-0.96	-0.123	0.687	0.514	0.918	0.022
**PSMC6-mRNA**	-0.587	0.182	-0.944	-0.23	0.666	0.52	0.852	0.00529
**TAB2-mRNA**	-0.601	0.252	-1.09	-0.108	0.659	0.468	0.928	0.0296
**CBLB-mRNA**	-0.618	0.254	-1.12	-0.119	0.652	0.461	0.921	0.0274
**TAX1BP1-mRNA**	-0.63	0.227	-1.07	-0.186	0.646	0.475	0.879	0.0134
**PSMD6-mRNA**	-0.639	0.262	-1.15	-0.125	0.642	0.449	0.917	0.0269
**PPM1A-mRNA**	-0.652	0.218	-1.08	-0.225	0.636	0.473	0.856	0.00859
**TOX4-mRNA**	-0.664	0.263	-1.18	-0.148	0.631	0.442	0.902	0.0225
**CREBBP-mRNA**	-0.689	0.194	-1.07	-0.309	0.62	0.477	0.807	0.00266
**PPP2R5E-mRNA**	-0.752	0.167	-1.08	-0.425	0.594	0.473	0.745	0.00036
**HGF-mRNA**	-0.794	0.339	-1.46	-0.128	0.577	0.364	0.915	0.0327
**LAMTOR3-mRN/**	-0.797	0.332	-1.45	-0.146	0.575	0.366	0.904	0.0289
**ATM-mRNA**	-0.804	0.238	-1.27	-0.338	0.573	0.414	0.791	0.00381
**PPP2R1B-mRNA**	-0.869	0.356	-1.57	-0.171	0.548	0.338	0.888	0.0266
**CCNC-mRNA**	-0.892	0.318	-1.52	-0.269	0.539	0.35	0.83	0.0127
**PPP2R5C-mRNA**	-0.915	0.271	-1.45	-0.383	0.531	0.367	0.767	0.00387
**PTPRJ-mRNA**	-0.988	0.347	-1.67	-0.308	0.504	0.315	0.808	0.0116
**IL18-mRNA**	-1.03	0.361	-1.73	-0.318	0.491	0.301	0.802	0.0118
**GRB10-mRNA**	-1.06	0.382	-1.81	-0.312	0.479	0.285	0.805	0.0135
**ITGA6-mRNA**	-1.09	0.39	-1.86	-0.328	0.469	0.276	0.797	0.0128
**LIFR-mRNA**	-1.09	0.498	-2.06	-0.114	0.47	0.239	0.924	0.0438
**ITPR1-mRNA**	-1.11	0.485	-2.06	-0.163	0.462	0.239	0.893	0.0355
**CSF3R-mRNA**	-1.14	0.418	-1.96	-0.325	0.452	0.256	0.798	0.0146
**ITPR2-mRNA**	-1.72	0.49	-2.68	-0.763	0.303	0.156	0.589	0.00285
**RGS16-mRNA**	-1.96	0.782	-3.49	-0.427	0.257	0.0888	0.744	0.0234
**PLCG2-mRNA**	-4.41	0.74	-5.86	-2.96	0.0471	0.0172	0.129	2.01e-05

Full blots, uncropped from FIGURE 5A

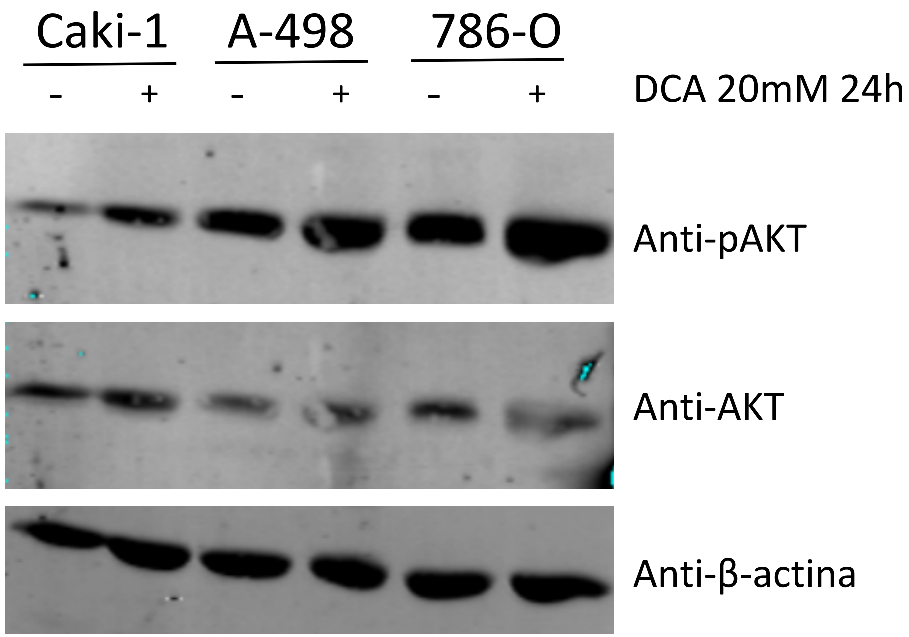

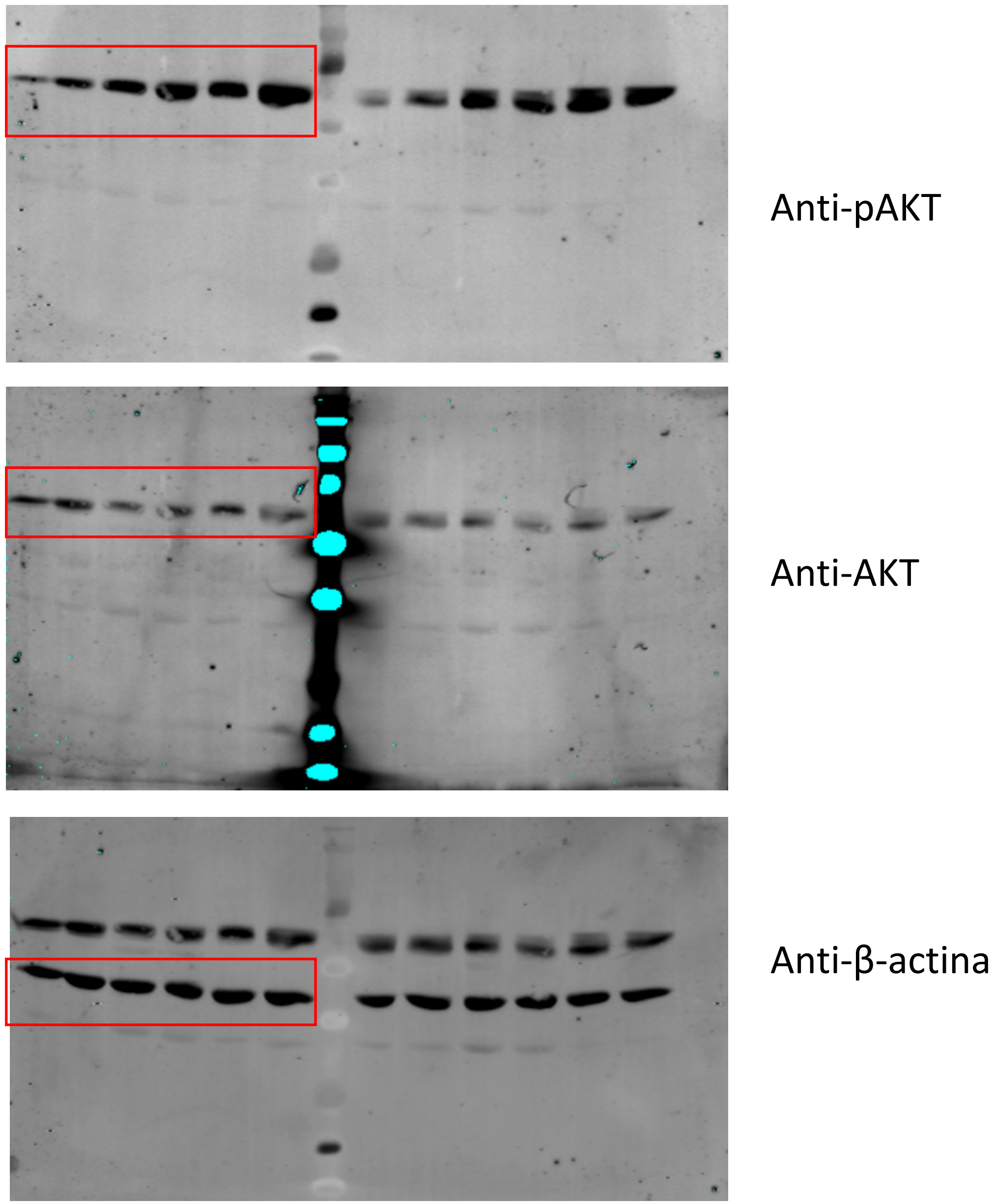

Supplement: Supplementary file 1 — Supplementary Information. [file 41598_2023_34087_MOESM1_ESM.pdf]
